# Clinical and Public Health Lessons from a Point-Source Polymicrobial Foodborne Outbreak: Implications for Vaccination of Vulnerable Populations

**DOI:** 10.3390/vaccines14090804

**Published:** 2026-09-12

**Authors:** Caterina Elisabetta Rizzo, Davide Anzà, Cristina Genovese, Dalila Sciacca, Salvatore Scondotto, Antonio Cristaldi, Vincenzo Restivo

**Affiliations:** 1 Department of Prevention, Local Health Authority of Enna, 94100 Enna, Italy; 2 Department of Medicine and Surgery, University of Enna Kore, 94100 Enna, Italy; davide.anza@unikore.it (D.A.); dalila.sciacca@unikorestudent.it (D.S.); salvatore.scondotto@unikore.it (S.S.); antonio.cristaldi@unikore.it (A.C.); 3 Department of Biomedical Sciences, Dental Sciences and Morpho-functional Imaging, University of Messina, 98100 Messina, Italy; crigenovese@unime.it

**Keywords:** foodborne outbreak, vulnerable populations, candidate vaccines, targeted immunization

## Abstract

Background: Foodborne outbreaks remain a major public health concern, particularly for immunocompromised individuals who experience a disproportionate burden of severe enteric disease. Although environmental prevention represents the cornerstone of food safety, outbreak investigations may also provide valuable evidence for identifying populations that could benefit most from future vaccination strategies. This study investigated a point-source foodborne outbreak in Southern Italy to describe clinical outcomes among exposed individuals, including an immunocompromised oncology patient, and to explore the potential relevance of host vulnerability for future enteric vaccine research. Methods: A retrospective cohort study combined with an environmental outbreak investigation was conducted following a restaurant-associated gastroenteritis cluster. Clinical data, epidemiological information, and laboratory findings were collected for all exposed individuals. Stool samples were analyzed using multiplex real-time polymerase chain reaction (RT-PCR), while microbiological and molecular analyses were performed on kitchen water samples collected during the official sanitary inspection. Results: Four of six exposed individuals developed acute gastroenteritis (overall attack rate: 66.7%; food-specific attack rate: 100%) within 24–48 h after consuming the same meal. Three previously healthy adults experienced prolonged symptoms lasting 15–21 days and subsequently developed post-infectious irritable bowel syndrome (PI-IBS) during follow-up. In contrast, a 43-year-old woman receiving cisplatin chemotherapy for cervical cancer developed severe dehydration requiring emergency hospitalization. Multiplex RT-PCR identified polymicrobial infection with *Campylobacter* spp., norovirus, and three diarrheagenic *Escherichia coli* pathotypes (EAEC, EPEC, and ETEC). Environmental investigation demonstrated contamination of the restaurant’s kitchen water supply with total coliforms (>80 CFU/100 mL), intestinal *Enterococci* (30 CFU/100 mL) and norovirus genogroups I and II, confirming the common source of exposure. Conclusions: This outbreak demonstrates how identical environmental exposure may result in markedly different clinical outcomes according to host immune status. While rigorous food safety measures remain essential, these findings may help develop hypotheses regarding populations that could be considered in future studies of enteric vaccination strategies. Outbreak investigations may therefore contribute not only to source identification and outbreak control but also to informing future vaccination strategies for vulnerable populations.

## 1. Introduction

Foodborne diseases remain a public health challenge globally, often manifesting as “point-source” outbreaks where a single contaminated vehicle leads to a cluster of cases within a narrow timeframe [1]. Latest data from the European Centre for Disease Prevention and Control (ECDC) show that the incidence of these diseases remains a top priority for health authorities across the continent [2]. In Europe, statistical data reveal a clear hierarchy of pathogens. Campylobacteriosis is still the most commonly reported zoonosis and is followed closely by Salmonellosis. The latter has been on a downward trend over the past decade due to more stringent controls in the poultry sector, but remains the main culprit in large-scale outbreaks, especially those associated with eggs and meat products [3]. Other pathogens such as Listeria monocytogenes present a different type of threat, with fewer cases but much more serious consequences, including hospitalization and high mortality rates among vulnerable populations [4]. Currently, in Italy, the national trend is largely similar to that of Europe, but with peculiar regionalities [5,6,7,8]. According to the integrated surveillance systems of the National Health Institute (Istituto Superiore di Sanità), the main etiologic agent of domestic outbreaks is consistently reported by Italian health authorities as *Salmonella* [9]. While most public health surveillance focuses on common pathogens like *Salmonella* or *Norovirus*, the emergence of polymicrobial outbreaks—where multiple bacterial and viral species are transmitted simultaneously—presents a more complex diagnostic and therapeutic landscape, imposing a considerable clinical and economic burden [10]. Although most infections are self-limiting in immunocompetent individuals, their clinical consequences may become substantially more severe in patients with impaired immune function, particularly those undergoing active oncologic treatment, the stakes are significantly higher [11]. The clinical severity of a foodborne infection is determined not only by the virulence of the pathogens but, more crucially, by the “fitness” of the host’s defenses [12]. In this outbreak, the index patient represented a textbook case of extreme physiological vulnerability. Patients undergoing platinum-based chemotherapy exist in a state of precarious immunological balance [13]. For these individuals, a common gastrointestinal insult can rapidly evolve into a systemic crisis due to two converging mechanisms: chemotherapeutic agents do not discriminate between malignant cells and the rapidly dividing cells of the gastrointestinal epithelium [14]. The resulting mucositis leads to a breakdown of the intestinal barrier—the “tight junctions” that normally prevent pathogens from entering the bloodstream [15]. This structural failure facilitates bacterial translocation and triggers an exaggerated inflammatory cascade.

Moreover, while the intestinal barrier is failing, the patient’s systemic defenses are simultaneously suppressed. A diminished immune system—often characterized by transient neutropenia or functional lymphocyte impairment—struggles to clear a high “microbial load.” [16]. This is particularly dangerous when the host is faced with a co-infection (e.g., *Campylobacter* and multiple *E. coli* pathotypes), where the pathogens may enhance each other’s pathogenicity [17,18]. Increased vulnerability to severe foodborne disease may result from a number of host- and exposure-related variables, such as immunosuppression brought on by medication, compromised intestinal barrier function, and environmental exposure conditions (Table 1).

Current preventive strategies rely predominantly on food safety measures, environmental monitoring, and official control systems based on Hazard Analysis and Critical Control Point (HACCP) principles [19]. Although these interventions have significantly reduced the incidence of foodborne diseases, they cannot completely eliminate exposure to enteric pathogens. Unexpected environmental failures, contaminated water supplies, asymptomatic food handlers, and complex food distribution networks continue to generate sporadic cases and point-source outbreaks [20]. These limitations emphasize the need for complementary preventive approaches capable of directly protecting susceptible hosts.

Vaccination represents one of the most effective public health interventions for preventing infectious diseases [21,22]. However, unlike other gastrointestinal pathogens, effective vaccines against several major causes of foodborne gastroenteritis remain unavailable or are still under clinical development [23]. Candidate vaccines against norovirus—including virus-like particle (VLP)-based formulations and emerging mRNA platforms—have shown encouraging immunogenicity and safety profiles in clinical trials [24,25,26], while several vaccine candidates targeting *Campylobacter* and enterotoxigenic *Escherichia coli* (ETEC) are currently under preclinical or early clinical evaluation [27,28,29]. Identifying groups at higher risk of severe illness is a crucial topic for future research as prospective enteric vaccines advance through development [30]. Outbreak investigations may offer supplementary real-world evidence on host vulnerability and illness severity that might guide the design and priority of future vaccine studies, but they are unable to prove vaccine efficacy or directly identify vaccine target populations.

Outbreak investigations provide a unique opportunity to address this knowledge gap. In-depth epidemiological studies enable the evaluation of host vulnerability, disease severity, transmission dynamics, and long-term clinical outcomes in real-world circumstances in addition to locating the source of infection and stopping transmission. Such data is especially useful for identifying target populations for upcoming immunization campaigns and guiding precision vaccination techniques meant to shield those most vulnerable to serious illness.

The aim of this work was to illustrate the variety of clinical outcomes within a precisely defined exposed cohort by integrating epidemiological, clinical, molecular, and environmental information from a naturally occurring point-source foodborne outbreak. These data are meant to generate hypotheses about host vulnerability and the representation of medically fragile populations in future investigations of potential vaccines in the context of enteric vaccine research.

## 2. Methods

### 2.1. Study Design and Outbreak Timeline

A retrospective cohort study and a point-source environmental outbreak report were conducted in March 2026 within the territorial jurisdiction of the Food Hygiene and Nutrition Service (SIAN) of the Local Health Authority of Enna, Italy. The epidemiological cohort comprised six working colleagues who shared a single lunch at a local restaurant. The clinical and epidemiological observation window spanned from the day of exposure (Day 0) up to 12 weeks post-exposure to rigorously evaluate both the acute recovery phases and the onset of long-term chronic gastrointestinal sequelae.

### 2.2. Study Population, Setting and Outbreak Cases Definitions

The setting was a public catering establishment (restaurant) located in Southern Italy. The study population included all individuals (*n* = 6) belonging to the specific occupational cohort who attended the shared lunch. The exposure event and the implicated public catering establishment were located in the Province of Enna, Sicily, Southern Italy. All environmental and public health investigations were conducted within the territorial jurisdiction of the Local Health Authority of Enna. According to the definitions described in Table 2, the outbreak comprised one laboratory-confirmed case (Patient 1), three probable cases (Patients 2–4), no possible cases, and two exposed non-cases (Patients 5–6).

Eligibility criteria for case inclusion required participants to have been present at the shared meal and to fulfill the clinical case definition. A clinical case of acute foodborne gastroenteritis was defined as any cohort member presenting with acute diarrhea (three or more loose or watery stools within a 24 h window), projectile vomiting, severe abdominal cramping, or systemic fever within an incubation window of 72 h post-exposure.

### 2.3. Clinical Data Collection and Molecular Diagnostic Assays

Detailed clinical histories, precise incubation timelines, symptom durations, and therapeutic intervention responses were documented for all cohort members. For the severely ill index patient, a diagnostic breakthrough was achieved using a stool sample collected immediately upon emergency hospital admission. The fecal specimen was subjected to rapid molecular diagnostic testing via multiplex real-time polymerase chain reaction (RT-PCR) panels. The multiplex assay targeted a comprehensive spectrum of enteric pathogens, including *Campylobacter* spp., *Salmonella* spp., *Shigella* spp., Norovirus genogroups 1 and 2, and specific virulent pathotypes of *Escherichia coli*.

### 2.4. Environmental Investigation and Laboratory Water Analysis

Immediately following the epidemiological notification, SIAN public health inspectors conducted an unannounced sanitary inspection of the restaurant’s kitchen facilities to identify structural hygiene failures, improper food handling practices, and compromised critical control points (CCPs). Water sampling was executed directly from the primary kitchen tap utilized by the staff for ingredient washing, food preparation, and cooking processes. Quantitative microbiological analyses for fecal indicator bacteria were executed by the competent official public health laboratory in strict compliance with current Italian regulatory standards (D.Lgs 18/2023) [31].

#### 2.4.1. Microbiological Analysis of Water Samples

The microbiological analyses included the quantification of coliform bacteria, *Escherichia coli* and intestinal enterococci. The enumeration of coliform bacteria and *E. coli* was performed according to UNI EN ISO 9308-1:2017 using the membrane filtration technique [32]. Briefly, 250 mL of each water sample was filtered through a sterile membrane filter with a nominal pore size of 0.45 μm. The membrane was subsequently placed on Chromogenic Coliform Agar (CCA; Lickson S.r.l., Palermo, Italy) and incubated at 36 ± 2 °C for 21–24 h. Following incubation, coliform bacteria and *E. coli* were differentiated and enumerated according to their characteristic chromogenic reactions. Furthermore, intestinal enterococci were assessed according to UNI EN ISO 7899-2:2003 using the membrane filtration technique as well [33]. According to this, 250 mL of each water sample was filtered through a sterile 0.45-μm pore-size membrane filter. The membrane was placed on Slanetz–Bartley agar (Lickson S.r.l., Palermo, Italy) and incubated at 36 ± 2 °C for 44 ± 4 h. Colonies exhibiting the characteristic red, brown, or pink coloration were considered presumptive intestinal enterococci. For confirmation, the membrane was transferred to pre-warmed Bile Aesculin Azide agar (Lickson S.r.l., Palermo, Italy) and incubated at 44 ± 0.5 °C for 2 h. Colonies exhibiting the characteristic brown-black coloration were considered confirmed intestinal enterococci. Results for coliform bacteria, *E. coli*, and intestinal enterococci were expressed as CFU per 100 mL.

#### 2.4.2. Norovirus Detection

For Norovirus detection, one-liter water samples were concentrated by polyethylene glycol (PEG) precipitation. According to this technique, PEG 6000 was added to the water samples to promote viral particle precipitation, followed by incubation under refrigerated conditions and centrifugation to recover the concentrated viral fraction. The resulting pellet was resuspended in a reduced volume, and viral RNA was extracted using the QIAamp Viral RNA Mini Kit (QIAGEN, Hilden, Germany), according to the manufacturer’s instructions. Extracted RNA was subsequently analysed by one-step reverse transcription quantitative real-time PCR (RT-qPCR) using the SuperScript III Platinum One-Step Quantitative RT-PCR System (Invitrogen, Carlsbad, CA, USA) according to the manufacturer’s instructions. The assay targeted the conserved ORF1/ORF2 junction region of the Norovirus genome. Separate RT-qPCR assays were performed for Norovirus genogroups GI and GII using genogroup-specific primers and TaqMan probes. For genogroup GI, the QNIF4 forward primer (5′-CGCTGGATGCGNTTCCAT-3′), NV1LCR reverse primer (5′-CCTTAGACGCCATCATCATTTAC-3′), and NVGG1p probe (5′-FAM-TGGACAGGAGAYCGCRATCT-TAMRA-3′) were used. For genogroup GII, the QNIF2 forward primer (5′-ATGTTCAGRTGGATGAGRTTCTCWGA-3′), COG2R reverse primer (5′-TCGACGCCATCTTCATTCACA-3′), and QNIFS probe (5′-FAM-AGCACGTGGGAGGGCGATCG-TAMRA-3′) were used. Appropriate positive and negative controls were included in each analytical run. Results were expressed qualitatively as positive or negative for Norovirus GI and GII.

### 2.5. Statistical Evaluation and Ethical Framework

Food-specific attack rates and overall cohort attack rates were calculated using standard epidemiological formulas. Descriptive statistical parameters, including medians, age ranges, and symptom duration blocks, were compiled. This containment and investigative activity was enforced as an official public health response under the authority of the regional prevention plan in accordance with the European law 625/2017 [34]. Data anonymization was maintained throughout and informed consent was obtained from all participants for the secure publication of their clinical data.

## 3. Results

### 3.1. Epidemiological Reconstruction and Cohort Attack Rates

**Figure 1:**
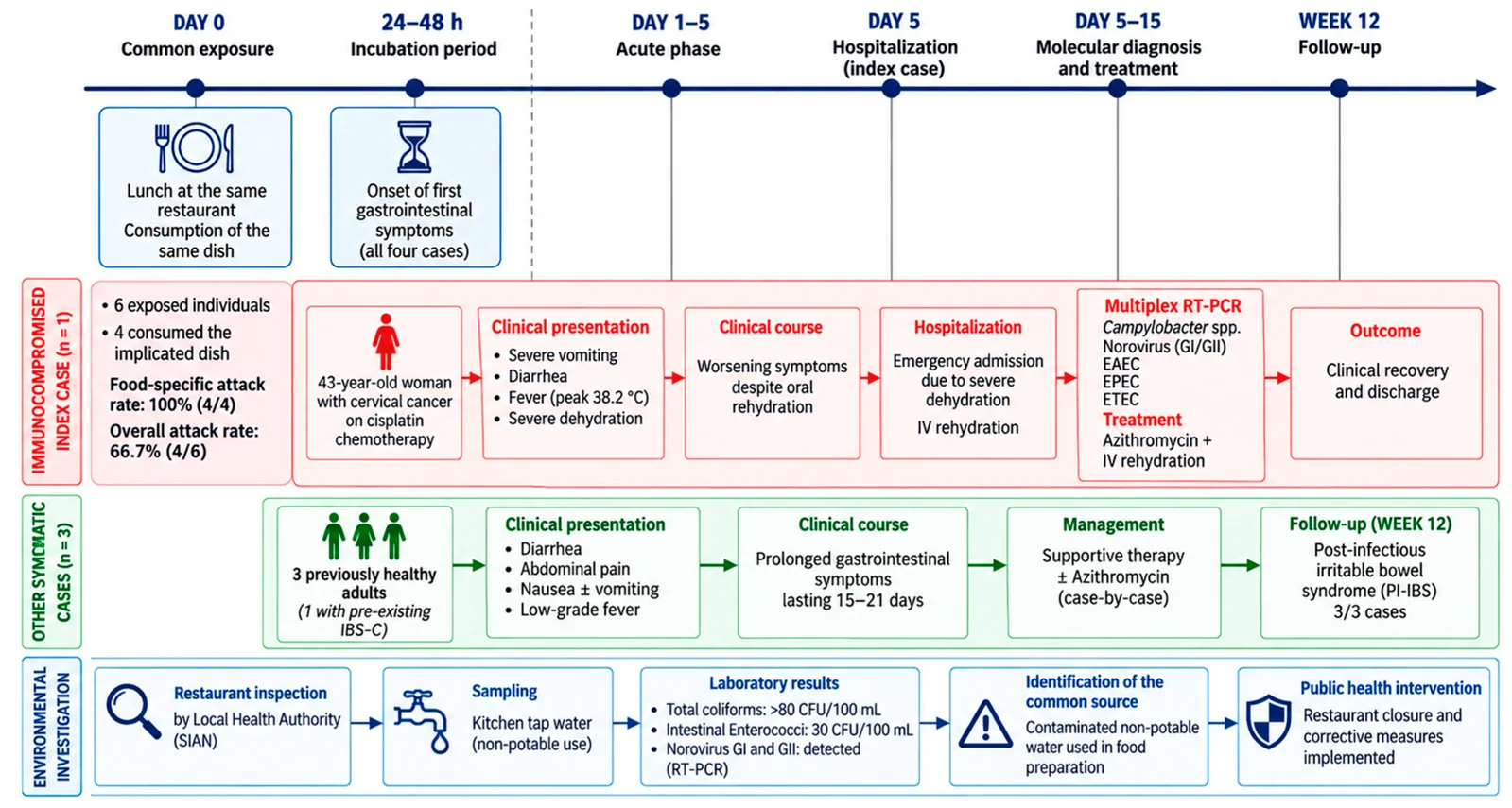
Epidemiological timeline, clinical outcomes and environmental investigation of the point-source foodborne outbreak.

The outbreak cohort comprised six working colleagues (age range: 27–44 years) who shared a single lunch at the implicated restaurant in March 2026 (Figure 1).

Detailed exposure tracking revealed that four out of six individuals consumed a specific dish consisting of basmati rice, zucchini, salmon, and soy sauce. The remaining two colleagues selected entirely different menu items and remained completely asymptomatic throughout the 12-week observation period. Consequently, the food-specific attack rate for the contaminated rice dish was 100% (4/4), while the overall cohort attack rate was 66.7% (4/6). The incubation period for all symptomatic individuals was highly uniform, ranging between 24 and 48 h post-ingestion. Patient 1 was a 43-year-old woman with International Federation of Gynecology and Obstetrics (FIGO) stage IIIC2 cervical cancer, for which she was receiving cisplatin-based chemotherapy [35]. Although the exposure occurred in Sicily, the index patient subsequently received emergency hospital care in Negrar, Verona, Northern Italy. This geographical difference reflects the location at which clinical care was provided and is unrelated to the location of the outbreak or environmental investigation. Within 24 h of consuming the meal, she experienced a fulminant onset of acute gastroenteritis characterized by projectile vomiting, high fever, and profuse, greenish watery diarrhea. Due to her chemotherapeutic status, oral rehydration was ineffective. On the fifth day of persistent symptoms, her worsening clinical status and severe dehydration required an emergency admission to the “Sacro Cuore Don Calabria” Hospital in Negrar, Italy.

Upon admission, clinical evaluation confirmed profound hypovolemic distress and marked asthenia. The diagnostic breakthrough was achieved via stool multiplex RT-PCR analysis, which revealed a massive polymicrobial co-infection consisting of: *Campylobacter* spp., Norovirus, and three distinct pathogenic pathotypes of *Escherichia coli*, specifically Enteroaggregative *E. coli* (EAEC), Enteropathogenic *E. coli* (EPEC), and Enterotoxigenic *E. coli* (ETEC). The patient required aggressive intravenous electrolytic rehydration and a targeted 3-day course of Azithromycin, which led to gradual clinical stabilization and subsequent resolution of the acute phase.

### 3.2. Acute Clinical Phase and Chronic Sequelae in the Healthy Cohort

The remaining three symptomatic individuals (Patients 2, 3 and 4) were females aged 27 to 44 years in good baseline health. Patient 2 reported a history of mild, diet-managed Irritable Bowel Syndrome with constipation (IBS-C), while Patients 3 and 4 had no significant prior gastrointestinal or systemic comorbidities. Despite their robust baseline immunity, the high microbial load delivered by the point-source vehicle triggered a severe and protracted clinical course. Within 24 to 48 h of exposure, all three patients experienced an acute onset of high-volume watery diarrhea, severe abdominal cramping, and crushing fatigue (severe asthenia) that caused total work absenteeism for nearly two weeks. Fever and total loss of appetite were uniformly reported during the initial 48 h. The acute diarrheal phase was remarkably prolonged, with gastrointestinal distress persisting for 15 to 21 days.

Regarding therapeutic interventions, Patient 2 was prescribed a 3-day course of Azithromycin following the molecular diagnosis of the index case, which induced a gradual reduction in stool frequency. Conversely, Patients 3 and 4 managed their symptoms exclusively with aggressive oral rehydration and commercial probiotics; this sub-group experienced the longest recovery times, highlighting a delayed clearance of the polymicrobial infection. Long-term longitudinal follow-up at 12 weeks revealed significant chronic dysfunction: all three women failed to return to their gastrointestinal baseline. They reported persistent visceral hypersensitivity, functional dyspepsia, severe “food phobia,” and situational anxiety regarding public dining, fulfilling the Rome IV diagnostic criteria for Post-Infectious Irritable Bowel Syndrome (PI-IBS) [36].

### 3.3. Environmental and Microbiological Water Investigations

The sanitary inspection conducted by public health inspectors at the restaurant highlighted critical structural hygiene failures. Structural monitoring of the kitchen water infrastructure revealed unmaintained water storage tanks and compromised internal plumbing. Quantitative microbiological analysis of water samples collected directly from the primary kitchen tap used for ingredient washing and food preparation confirmed a catastrophic breach of official potability standards.

These environmental findings confirmed that the non-potable water supply functioned as the primary vehicle for widespread cross-contamination, exposing safe raw ingredients (rice, vegetables, and salmon) to high-titer fecal and viral pathogens during the final preparation and washing stages. Immediate administrative closure of the restaurant and judicial referrals were executed by the local health authority to interrupt the active transmission chain.

## 4. Discussion

The conventional goals of foodborne outbreak investigations are to stop the transmission of infection, trace the source and vehicle of infection and stop further cases. Tightly defined common-source exposure events, however, might also offer a chance to investigate the variation in clinical outcomes among those exposed to comparable epidemiological conditions. Given that host-related factors can significantly impact the clinical manifestation and severity of enteric infections, this perspective is particularly valuable when medically fragile patients are involved. In these terms, this study combines environmental, molecular, clinical, and epidemiological data from a single source foodborne outbreak, aligning with broader regional data [37]. The results indicate how environmental contamination and host vulnerability interact, and they also emphasize how crucial it is to combine source management with close clinical monitoring of those who are more likely to experience serious consequences. Under existing European Union regulations, the scheduling of official controls is primarily periodic, a structural approach that precludes the continuous monitoring of epidemiological trends [38]. This lack of longitudinal oversight is particularly detrimental to the protection of immunocompromised cohorts, whose heightened susceptibility to opportunistic pathogens demands a more responsive and real-time assessment of environmental risks [39]. Furthermore, the intermittent nature of these regulatory inspections contributes to a significant underestimation—or “underreporting”—of waterborne and foodborne illnesses [40]. In a restaurant scenario, the identification of fecal indicator organisms in water used for food preparation is especially important since contaminated water can serve as a source of secondary contamination of food, utensils, and surfaces that come into contact with food in addition to acting as a direct exposure vehicle. Because many infections in fragile populations may present as sporadic incidents rather than large-scale outbreaks, they often remain undetected by routine, interval-based monitoring. To address this evidentiary gap, it is imperative to shift toward more adaptive planning strategies that incorporate the surveillance of indirect proxy indicators. For instance, integrating real-time data on Emergency Department (ED) admissions for gastrointestinal distress could serve as an early-warning signal [41]. Utilizing such indirect parameters would facilitate a more proactive epidemiological posture, allowing for the detection of subtle shifts in pathogen prevalence that current periodic controls are ill-equipped to identify.

### 4.1. Food Safety and Vaccination as Complementary Prevention Strategies

From a preventive standpoint, foodborne outbreaks emphasize the evolving landscape of such threats. While traditional pathogens like *Salmonella* continue to be a primary focus, the emergence of polymicrobial infections in fragile populations necessitates a shift in prevention tools [42]. Nevertheless, as demonstrated by the present study, these measures cannot completely eliminate the risk of accidental exposure resulting from infrastructural failures, contaminated water supplies or unforeseen breaches in hygiene practices. This concept is particularly relevant for individuals with impaired immune function.

While environmental interventions reduce the probability of exposure, they cannot directly modify host susceptibility. Consequently, prevention in fragile populations should increasingly be viewed as a dual strategy that combines environmental risk reduction with host-directed protection (Table 3). The clinical trajectory observed in these cases further aligns with documented evidence regarding the heightened vulnerability of immunosuppressed populations to atypical or prolonged infections [43]. Recent literature consistently demonstrates that in patients with primary or secondary immunodeficiencies, the clinical presentation of common pathogens such as *Salmonella* or *Campylobacter* is frequently characterized by increased severity, a higher propensity for systemic dissemination and a protracted duration of pathogen shedding [44].

### 4.2. Public Health Implications

Despite the substantial global burden of foodborne gastroenteritis, vaccines remain unavailable for several of the most common enteric pathogens [45]. Norovirus vaccine development has progressed considerably during the past decade, with virus-like particle (VLP)-based vaccines and more recently mRNA platforms demonstrating encouraging immunogenicity and acceptable safety profiles in clinical studies [46]. Similarly, several vaccine candidates targeting *Campylobacter* spp. and enterotoxigenic *Escherichia coli* (ETEC) are currently undergoing preclinical or early clinical evaluation. Future vaccination against major enteric pathogens should therefore not be considered an alternative to food safety but rather a complementary preventive approach capable of reducing disease severity among individuals at highest clinical risk [47,48,49,50].

Furthermore, the polymicrobial nature of this outbreak highlights the complexity of enteric disease ecology and suggests that future multivalent or combination vaccine approaches may become increasingly relevant, particularly for populations exposed to multiple gastrointestinal pathogens [51,52,53,54].

Rather than adopting universal vaccination strategies for all enteric pathogens, future immunization programs may prioritize individuals with substantially increased susceptibility to severe disease [55], including patients undergoing chemotherapy, recipients of solid-organ or hematopoietic stem cell transplantation, individuals receiving biological immunosuppressive therapies, and other medically fragile populations [56,57,58]. The idea that host features affect the severity of enteric infections is supported by the diverse clinical outcomes seen after a shared exposure [59]. These findings indicate that medically fragile people should be sufficiently represented in future studies assessing potential enteric vaccines, even when they cannot prove the benefit of immunization.by demonstrating that identical environmental exposure may generate profoundly different clinical outcomes depending on host characteristics [60]. Outbreak investigations therefore represent valuable real-world models for identifying those populations in whom future vaccines could provide the greatest clinical benefit [61,62].

### 4.3. Strengths and Limitations

The integration of epidemiological, clinical, molecular, and environmental findings within a precisely defined point-source exposure, together with long-term clinical follow-up, is this investigation’s main strength. However, there are a few restrictions to take into account. First, no inferential statistical analyzes were necessary because the cohort was extremely small (*n* = 6), which reflected the limited scope of the exposure event. Second, the more severe clinical course shown in this patient cannot be causally linked to immunological status or extrapolated to immunocompromised populations because just one participant was immunocompromised. Third, it is not possible to assume that all outbreak cases have the same combination of pathogens because extensive molecular testing was available for the index patient but not for all symptomatic individuals.

## 5. Conclusions

This point-source outbreak demonstrates that environmental water quality is a fundamental, mandatory pillar of food security and a crucial critical control point (CCP) within the public catering sector. A single infrastructural failure in a restaurant’s water system can distribute a resilient, high-titer polymicrobial cocktail capable of inducing chronic gastrointestinal sequelae in healthy individuals and triggering life-threatening systemic crises in fragile hosts. For oncological patients, such preventable hygiene failures transcend simple gastrointestinal distress, forcing the dangerous suspension of vital, life-saving chemotherapeutic regimens. Although the present investigation was not designed to evaluate vaccine efficacy, it provides clinically relevant evidence regarding the populations most likely to benefit from future immunization strategies. The disproportionate severity observed in the chemotherapy patient supports the concept that individuals with treatment-induced immunosuppression could represent priority candidates for enteric vaccination once safe and effective vaccines become available.

## Figures and Tables

**Table 1:** Clinical risk factors for severe foodborne illness along with possible potential pathophysiological implications.

Clinical Impact in This Case	Mechanism of Action	Risk Factor
Accelerated onset of symptoms and increased gut permeability.	Induction of severe GI mucositis and epithelial denudation.	Active Cisplatin Therapy
Refractory diarrhea and failure of standard probiotic treatment.	Combined exposure to bacterial and viral enteropathogens	Concurrent detection of multiple enteric pathogens
Prolonged bacterial shedding and risk of systemic translocation.	Reduced macrophage and T-cell response to enteric pathogens.	Immunosuppression
Rapid progression to severe dehydration and metabolic imbalance.	Chemotherapy-induced renal stress combined with fluid loss.	Electrolyte Imbalance
Continuous exposure to pathogens during food preparation/washing.	Point-source exposure to high-titer fecal coliforms	Water Supply Contamination

**Table 2:** Epidemiological case definitions applied in the outbreak investigation.

Laboratory Criteria	Epidemiological/Exposure Criteria	Clinical Criteria	Case Classification
Detection of ≥1 enteric pathogen by molecular testing	Participation in the shared meal and consumption of the implicated dish	Acute gastroenteritis compatible with the outbreak case definition within 72 h after exposure	Confirmed case
No microbiological confirmation available	Consumption of the implicated dish and epidemiological link to the confirmed case/common source	Acute gastroenteritis compatible with the outbreak case definition within 72 h after exposure	Probable case
No microbiological confirmation available	Epidemiological association with the event, but consumption of the implicated dish not confirmed	Compatible gastrointestinal symptoms temporally associated with the outbreak	Possible case
Not required	Participation in the shared meal	No gastrointestinal symptoms during the predefined observation period	Exposed non-case

**Table 3:** Potential implications of the present outbreak investigation for future enteric vaccination strategies.

Potential Implication for Vaccination	Evidence Generated by Outbreak Investigation	
Defines the epidemiological context in which vaccination may reduce disease burden.	Identification of the contaminated food vehicle and environmental source	Exposure assessment
Supports prioritization of pathogens for future vaccine development.	Detection of bacterial and viral enteropathogens	Pathogen identification
Identifies populations most likely to benefit from vaccination.	Comparison between healthy and immunocompromised individuals	Clinical characterization
Expands vaccine endpoints beyond prevention of acute infection.	Hospitalization, prolonged illness and PI-IBS	Outcome assessment
Highlights vaccination as an additional preventive layer within integrated food safety strategies.	Environmental remediation and outbreak control	Public health response

## Data Availability

The clinical, epidemiological, and environmental data supporting the findings of this outbreak investigation are derived from official institutional records of the Food Hygiene and Nutrition Service of the Local Health Authority of Enna (ASP Enna, Sicily, Italy). Anonymized source data can be requested from the corresponding author upon reasonable scientific justification.
